# Multifocal Stroke and Gait Ataxia in the Elderly: A Peculiar Case of Meningovascular Neurosyphilis With Posterior Circulation Infarction

**DOI:** 10.7759/cureus.108506

**Published:** 2026-05-08

**Authors:** Kyle Sharron, Manveer Sandhu, Damin Singh, Praful R Pendyala, Gabrielle Brini, Syed Nasir

**Affiliations:** 1 Neurology, Touro College of Osteopathic Medicine, Middletown, USA; 2 Neurology, Garnet Health Medical Center, Middletown, USA; 3 Neurology, Chalmeda Anand Rao Institute of Medical Sciences, Karimnagar, IND; 4 Radiology, Touro College of Osteopathic Medicine, Middletown, USA

**Keywords:** geriatrics, meningovascular neurosyphilis (mvs), neurology, neurosyphillis, pca infarction

## Abstract

An 83-year-old male presented with a months-long ataxic gait and recent frequent falls. Initial evaluation revealed ventriculomegaly and acute ischemic infarction to the right cerebellar and occipital regions in addition to severe cervical stenosis. Syphilis serology with reflex returned positive, and neurosyphilis was confirmed on cerebrospinal fluid with a reactive Venereal Disease Research Laboratory test, lymphocytic pleocytosis, and elevated protein. The patient was put on a two-week course of intravenous penicillin G while completing inpatient physical rehabilitation and demonstrated significant improvement. This case highlights the importance of considering neurosyphilis as a differential in the elderly with new-onset gait disturbance and first-time stroke without a contributory past medical history. This report underscores the emergence of neurosyphilis in elderly populations and the challenge in correctly identifying cases in this demographic, considering its reputation as a “great imitator” of other neurocognitive disorders.

## Introduction

Neurosyphilis is a manifestation of latent or tertiary syphilis infections, often in the setting of chronic, unresolved inoculation that results in invasion of the central nervous system (CNS). Between 2001 and 2021, rates of syphilis increased in North America from 2.1 per 100,000 to 17.6 per 100,000 [1]. Once considered rare due to empiric antibiotic use, neurosyphilis is now re-emerging globally, particularly in older adults.

Clinical presentations of neurosyphilis can vary widely, and this contributes to its elusiveness, known as the great imitator. This overlap with several neurodegenerative disorders makes the diagnosis and recognition of neurosyphilis in the elderly population especially challenging [2,3].

Failure of the immune system to clear syphilis from the cerebrospinal fluid (CSF) is thought to result in various neurologic complications, including meningovascular syphilis, a manifestation of syphilis that results in thrombosis and infarction due to endarteritis of the CNS [4]. While syphilitic arteritis can affect any part of the cerebral vasculature, the middle cerebral artery territory accounts for the largest proportion of cases. However, substantial evidence suggests that the posterior circulation, including the pons, is also significantly affected. Additionally, Daza-Ovalle et al. reported a fatal case of vertebrobasilar occlusion due to meningovascular syphilis, further emphasizing the impact on the posterior circulation [5]. Recovery from neurosyphilis has been reported to be quite variable and oftentimes dependent on early access to antibiotic therapy to halt the disease’s progression [6].

We present the case of an elderly man with progressive gait instability and recurrent falls who was ultimately determined to have neurosyphilis in the setting of severe cervical stenosis and acute cerebral infarcts. This case highlights the ambiguity of the disease and the wide manifestations of potential symptoms. Through this case, we emphasize the importance of considering neurosyphilis as a differential in the evaluation of neurologic decline in the elderly demographic and in the setting of various other strong differentials.

## Case presentation

**Figure 1:**
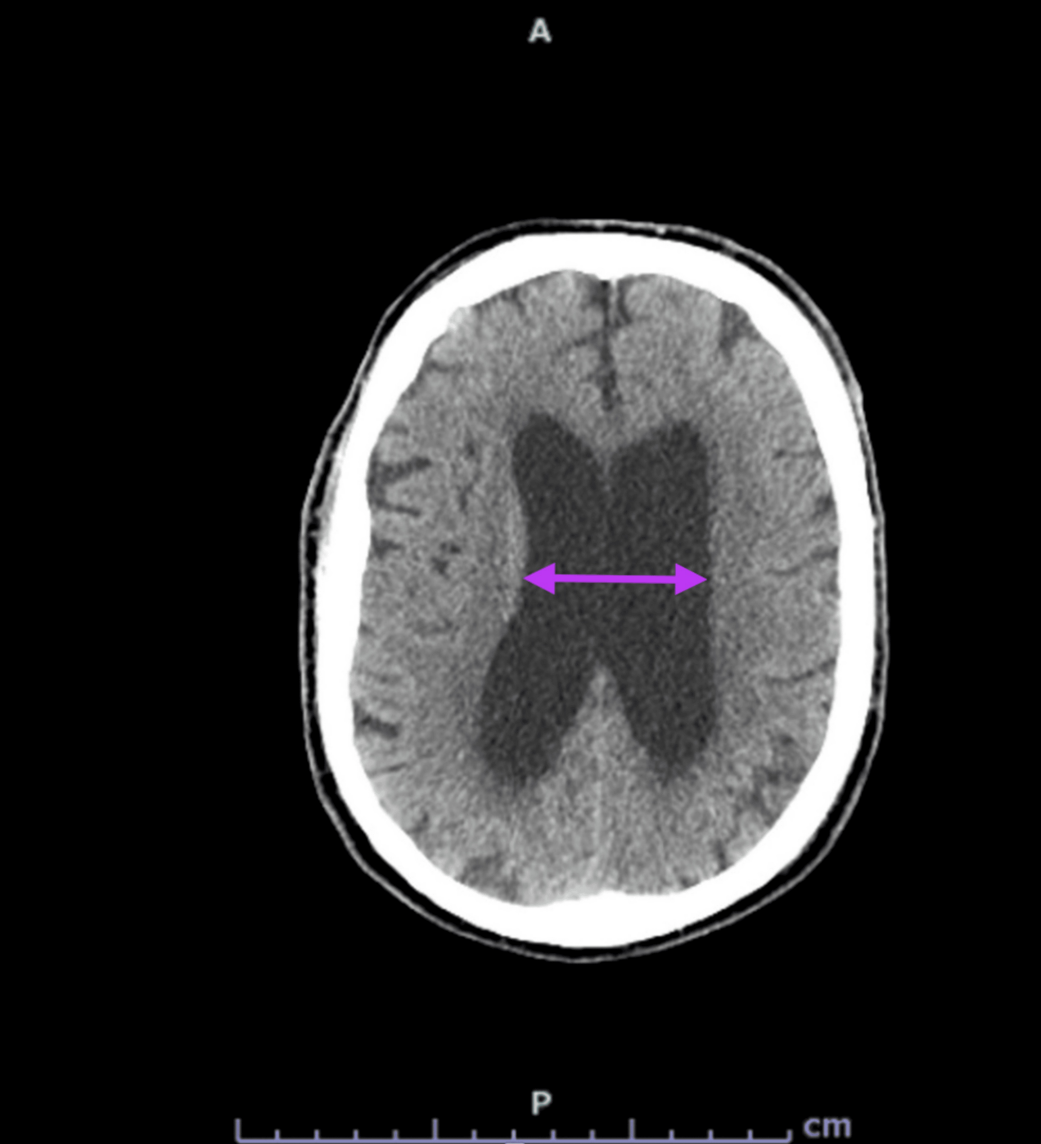
Non-contrast CT of the brain demonstrating ventriculomegaly. The arrows depict ventricular enlargement.

An 83-year-old male with no significant past medical history presented to the emergency department with a several-month history of progressively worsening gait and generalized malaise, with a single episode of urinary incontinence. Before this decline, the patient was independent in all activities of daily living and used a cane intermittently. Upon admission, the patient was noted to be hypotensive, had a low-grade fever (100.1°F/37.8°C), and mild leukocytosis (12.1 K/µL). Importantly, no antibiotics were administered between admission and lumbar puncture, ensuring unconfounded CSF analysis. Neurology was consulted for gait abnormality. On initial evaluation, the patient was at his cognitive baseline: alert and oriented with no deficits of attention, memory, language, or reasoning. Cranial nerves II-XII were intact. There were signs of upper motor neuron involvement, including a distinct shuffling gait, hyperreflexia in both upper and lower extremities, positive Hoffmann sign, and ankle clonus. Non-contrast CT of the brain revealed ventriculomegaly disproportionate to the mild cortical volume loss (Figure 1), raising concern for normal pressure hydrocephalus.

**Figure 2:**
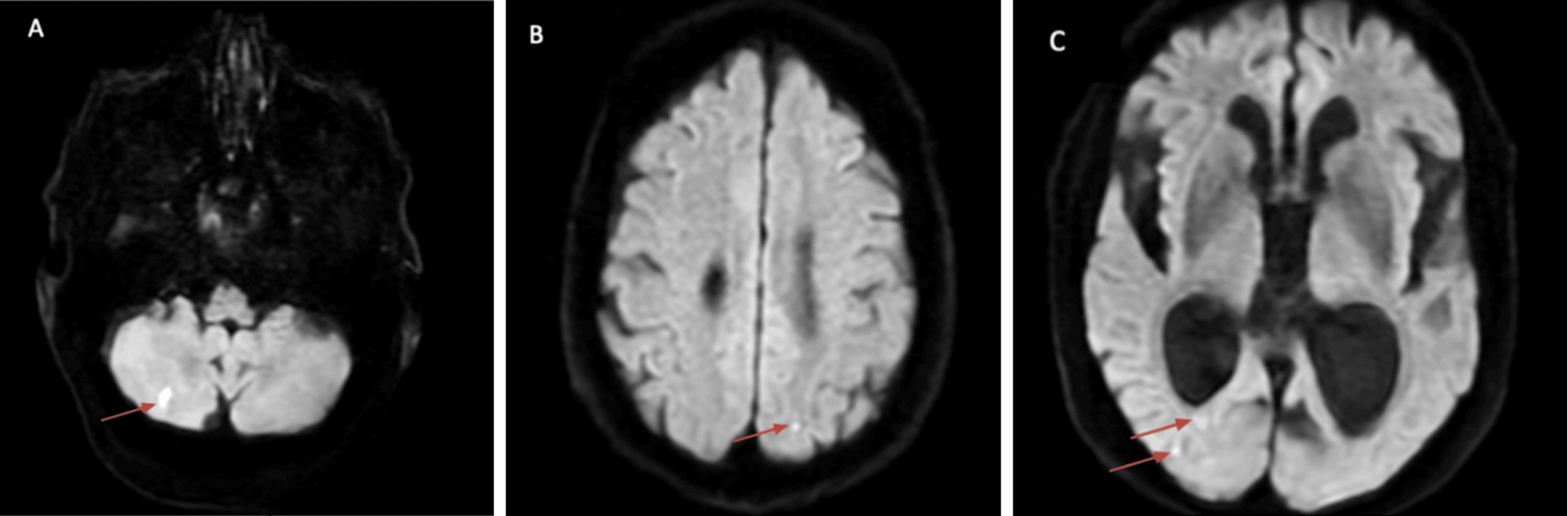
MRI of the brain without contrast demonstrating acute infarcts. (A) Acute right cerebellar infarct. (B) Acute left occipital infarct. (C) Acute right occipital infarcts.

**Figure 3:**
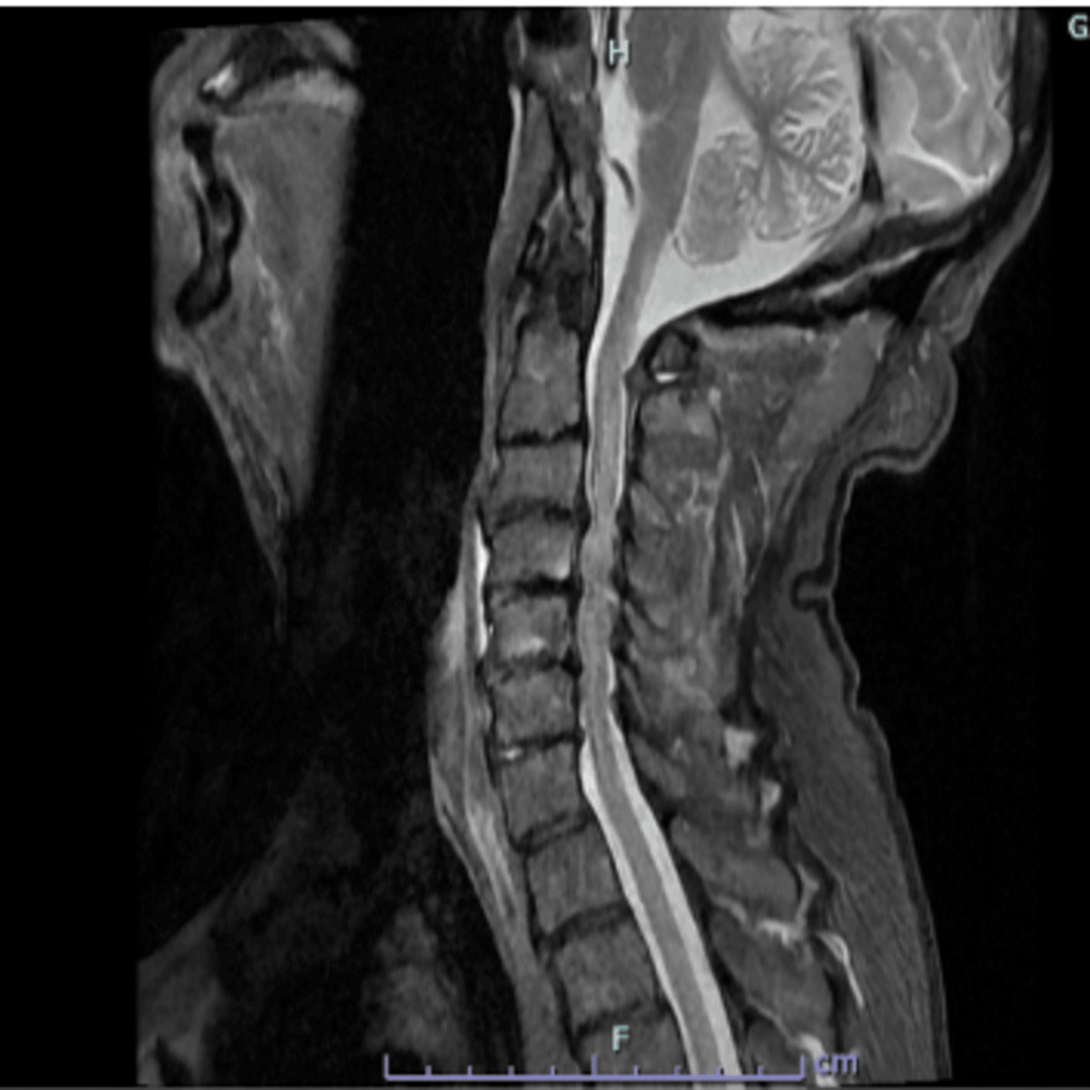
MRI of the cervical spine without contrast demonstrating central canal stenosis at levels C3-C4 and C4-C5.

Both MRI of the brain and cervical spine were obtained. The MRI of the brain without contrast showed an acute, non-hemorrhagic right cerebellar cerebrovascular accident (CVA); however, it was unlikely to be the sole cause of gait ataxia given the small stroke burden. Additionally, restricted diffusion was observed in the right and left occipital lobes (Figures 2a-2c). The MRI of the cervical spine was equally striking, revealing severe central canal stenosis at C3-C4 and C4-C5, indicative of cervical myelopathy (Figure 3). A CT angiography of the head and neck was unremarkable, showing no obvious vasculitis, aneurysm, or flow-limiting stenosis.

With two major structural pathologies, i.e., acute stroke and severe cervical myelopathy, overwhelming the clinical picture, the initial diagnostic and management plan was complex. Neurosurgery was consulted for the severe stenosis; however, the patient declined surgical intervention. Antithrombotic therapy and high-intensity statin were initiated at the same time as standard stroke prevention.

Crucially, given the initial systemic signs of inflammation (fever and leukocytosis), a broader infectious workup was initiated, including a rapid plasma reagin (RPR) test. Upon further history, the patient disclosed a single unprotected sexual encounter before his 60-year monogamous marriage, during his time in Vietnam. The RPR serology was reactive, and subsequent blood treponemal-specific antibody testing was positive. In conjunction with an Infectious Disease consultation, a lumbar puncture (LP) was performed to rule out neurosyphilis.

CSF analysis showed an elevated white blood cell count with lymphocytic pleocytosis and elevated protein, suggesting an active inflammatory process (Table 1). The diagnosis of neurosyphilitic meningitis was confirmed by a positive treponema indirect fluorescent antibody (IFA) in the CSF. HIV 1/2 Ag/Ab testing was negative, illustrating the absence of any HIV-associated disease acceleration or confounding CNS pathology. The patient was immediately started on an aggressive 14-day course of intravenous (IV) aqueous penicillin G.

**Table 1:** CSF analysis demonstrating corrected leukocytic pleocytosis with lymphocytic predominance, elevated protein, and positive VDRL confirmatory of neurosyphilis infection. *: Corrected CSF WBC using the standard formula = Measured CSF WBC - (blood WBC × CSF RBC/Blood RBC). CSF = cerebrospinal fluid; VDRL = Venereal Disease Research Laboratory; WBC = white blood cell; RBC = red blood cell

	Result	Reference range	Interpretation
Appearance	Clear, colorless	–	Normal
RBC	325	0 cells	Elevated
WBC	10 cells	0–5 cells	Leukocytosis
Cell predominance	97% lymphocytes	–	Lymphocytic pleocytosis
Protein	54 mg/dL	15–45 mg/dL	Elevated
Glucose	54 mg/dL	40–70 mg/dL	Normal
VDRL	1:4 titer	Non-reactive	Positive for *Treponema pallidum*

Following initiation of antibiotic therapy, the patient was transferred to the acute rehabilitation unit. Over the three-month period of gait disturbance leading to admission, the patient’s gait had evolved into an ataxic, cerebellar predominant pattern. With IV penicillin G and intensive inpatient physical therapy, he demonstrated progressive and clinically meaningful improvement in gait and lower extremity strength. Functional gains were reflected by an improvement in modified Rankin Scale score from 4 (at time of admission) to 3 (two weeks following the completion of antibiotic therapy), highlighting both the treatable nature of neurosyphilis and the importance of timely identification. At the time of discharge from rehabilitation, he was ambulating with minimal assistance, and at outpatient follow-up completed the prior fall, he was walking independently without any assistive device, representing a return to his premorbid functional baseline. The patient is scheduled to follow up as an outpatient with Neurology and PM&R, but has not been seen yet.

Montreal Cognitive Assessment (MoCA) was administered at the end of the treatment course, yielding a score of 22/30. This falls below the standard cutoff of 26/30 typically used to screen for mild cognitive impairment. It is important to note that MoCA performance is influenced by educational attainment, and education-adjusted normative data should be applied when interpreting borderline scores. Without a cognitive baseline, it remains difficult to determine the degree to which the observed impairment reflects accelerated neurodegeneration driven by neurosyphilis versus age-related cognitive decline. Longitudinal reassessment is planned.

## Discussion

This case highlights the diagnostic complexity of evaluating neurological deterioration in an elderly patient with multiple coexisting conditions capable of producing overlapping symptoms. Gait ataxia and urinary incontinence initially raised concern for normal pressure hydrocephalus, supported by ventriculomegaly on neuroimaging. Concurrently, chronic severe cervical stenosis at levels C3-C4 and C4-C5 with associated myelopathy represented a compelling alternative explanation, given its potential to produce gait dysfunction, falls, urinary symptoms, and brisk reflexes. Additionally, acute infarcts in the cerebellar and occipital lobes within the posterior circulation provided yet another plausible source of gait instability and visual disturbance.

Despite these competing diagnoses, several atypical features prompted reconsideration of the initial differential. The subacute progression of symptoms, the presence of a low-grade fever, and mild leukocytosis were not fully consistent with the natural history of chronic cervical stenosis or isolated posterior circulation stroke. These inflammatory features raised suspicion for an underlying infectious or immune-mediated process. Further nontreponemal infectious workup, including respiratory influenza viral testing and CSF meningitis/encephalitis panel polymerase chain reaction, returned negative. Serologic evaluation demonstrated positive RPR and confirmatory fluorescent treponemal antibody absorption results for syphilis, warranting CSF analysis. This proved diagnostic, demonstrating lymphocytic pleocytosis and elevated protein with a positive CSF-Venereal Disease Research Laboratory (VDRL) and positive CSF-IFA consistent with neurosyphilis. CSF treponemal assays, such as IFA, have high sensitivity but poor specificity, making his positive IFA result supportive rather than confirmatory. Nontreponemal CSF testing (VDRL) is highly specific but only moderately sensitive, whereby his positive CSF-VDRL was confirmatory [4].

An important contextual factor in this case is the patient’s social history. Although he reported many decades of monogamy with his wife, he disclosed remote unprotected sexual encounters during military deployment decades ago. This detail underscores the remarkable latency and clinical variability of syphilis, which can remain asymptomatic for decades before manifesting with neurological involvement. Such an extended interval between exposure and presentation is well-documented, and it reinforces the diagnostic challenge syphilis poses, particularly in older adults in whom late-stage manifestations may mimic numerous other conditions.

The tempo of the patient’s decline, marked by subacute worsening gait imbalance, was incongruent with chronic degenerative cervical stenosis. Similarly, the embolic-appearing posterior circulation infarcts occurred despite the absence of conventional vascular risk factors and with a negative echocardiogram, reducing the likelihood of a primary cardioembolic etiology. Stroke is the presenting manifestation in approximately 10% of neurosyphilis patients, reinforcing its relevance in this case [7]. A previous case reported a fatal case of vertebrobasilar occlusion due to meningovascular neurosyphilis. Taken together, these findings supported meningovascular syphilis as the unifying diagnosis capable of explaining the patient’s multifaceted neurological symptoms. Meningovascular neurosyphilis results from an obliterative endarteritis causing ischemia and stroke, often in the context of a chronic meningeal inflammatory process, and is well recognized for its ability to masquerade as a wide array of neurological disorders.

The patient’s significant clinical improvement following a 14-day course of IV penicillin G, paired with structured inpatient rehabilitation, further substantiated the diagnosis. Functional gains were reflected by an improvement in modified Rankin Scale score from 4 (pre-treatment) to 3 (post-treatment), highlighting both the treatable nature of neurosyphilis and the importance of timely identification.

This case emphasizes the continued relevance of neurosyphilis as a diagnostic consideration in geriatric neurology. As “the great imitator,” neurosyphilis can closely resemble degenerative spine disease, normal pressure hydrocephalus, and vascular pathology, delaying recognition, particularly when multiple comorbidities coexist. Maintaining a high index of suspicion is essential in older patients presenting with subacute neurological decline, atypical stroke patterns, or inflammatory features that are not fully explained by common geriatric conditions. Early diagnosis and treatment are critical to preventing irreversible neurological injury and optimizing recovery.

## Conclusions

This case illustrates the diagnostic complexity of neurosyphilis in an elderly patient presenting with overlapping neurological pathologies, including acute cerebellar and occipital infarcts, severe cervical myelopathy, and ventriculomegaly. The concurrent structural findings initially obscured the underlying infectious etiology, underscoring the risk of anchoring bias when compelling radiologic abnormalities dominate the clinical picture. A broad infectious workup, including syphilis serology and CSF analysis, proved essential in establishing the correct diagnosis and initiating timely treatment. The patient’s meaningful functional recovery following a 14-day course of IV penicillin G, combined with targeted rehabilitation, reinforces that elderly patients with neurosyphilis can achieve significant improvement when the diagnosis is not delayed. As syphilis rates continue to rise across all demographic groups, clinicians must maintain a low threshold for syphilis screening in older adults presenting with unexplained neurologic decline, even in the absence of traditional risk factors.
